# Temporal changes in tibiofemoral relationship following anterior cruciate ligament injury: Implications for rotational dynamics and clinical outcomes

**DOI:** 10.1002/jeo2.70014

**Published:** 2024-09-09

**Authors:** Shinichiro Okimura, Tomoyuki Suzuki, Yasutoshi Ikeda, Kousuke Shiwaku, Kodai Hamaoka, Kazushi Horita, Atsushi Teramoto

**Affiliations:** ^1^ Department of Orthopaedic Surgery Obihiro Kyokai Hospital Obihiro Hokkaido Japan; ^2^ Department of Orthopaedic Surgery Sapporo Maruyama Orthopaedic Hospital Sapporo Hokkaido Japan; ^3^ Department of Orthopaedic Surgery Sapporo Medical University School of Medicine Sapporo Hokkaido Japan

**Keywords:** ACL, anterior tibial translation, chronicity of ACL deficiency, tibial rotation

## Abstract

**Purpose:**

This study aims to elucidate changes in the tibiofemoral relationship over time following anterior cruciate ligament (ACL) injury, and investigate the correlation between the tibiofemoral relationship and patient‐related outcome measures (PROMs).

**Methods:**

Overall, 203 primary ACL reconstructions were performed using autologous hamstring grafts. Medial and lateral anterior tibial translation (ATT) in the sagittal plane and tibial tubercle‐trochlear groove (TT‐TG) distance in the axial plane were measured using pre‐operative magnetic resonance imaging and post‐operative computed tomography. The difference between pre‐operative and post‐operative values for each parameter was calculated: ΔMesdial ATT, ΔLateral ATT and ΔTT‐TG distance. The correlation between each calculated value and the time elapsed since ACL injury, and the correlation between each calculated value and PROMs—evaluated using the Knee Injury and Osteoarthritis Outcome Score (KOOS)—were assessed.

**Results:**

Sixty‐four patients were enroled. Medial ATT, lateral ATT and TT‐TG distance were significantly different pre‐operatively compared to post‐operative values, with the tibia translating anteriorly and rotating internally relative to the femur. ΔMedial ATT, ΔLateral ATT and ΔTT‐TG distance were 1.6, 8.8 and −4.8 mm, respectively. A negative correlation was observed between the ΔTT‐TG distance and the time elapsed since the injury (*r* = −0.44, *p* < 0.01). No correlation was found between ΔMedial ATT and the time elapsed since the injury, nor between ΔLateral ATT and the time elapsed since the injury. Neither the ΔMedial ATT, ΔLateral ATT, nor ΔTT‐TG distance correlated with the pre‐operative or post‐operative KOOS subscale scores.

**Conclusions:**

The tibia underwent internal rotation relative to the femur over time following ACL injury, highlighting the importance of assessing rotational changes in ACL‐injured knees.

**Level of Evidence:**

Level Ⅲ.

**Abbreviations**
ACL: anterior cruciate ligament
ACLR: ACL reconstruction
ATT: anterior tibial translation
CT: computed tomography
KOOS: Knee Injury and Osteoarthritis Outcome Score
MRI: magnetic resonance imaging
PROM: patient‐related outcome measure
TT‐TG: tibial tubercle‐trochlear groove

## INTRODUCTION

Anterior cruciate ligament (ACL) injuries are the most prevalent knee ligament injuries. The ACL plays a crucial role in maintaining a normal tibiofemoral relationship, preventing anterior translation and preserving knee rotational stability. Previous studies have highlighted distinctions in the tibiofemoral relationship between knees with ACL deficiency and those with intact ACLs [16, 29]. Notably, prolonged ACL deficiency can lead to an abnormal tibiofemoral relationship, potentially resulting in meniscus injury, cartilage damage and osteoarthritis progression [16].

It is widely recognized that ACL deficiency leads to a change in the tibiofemoral relationship within the knee joint [20]. The chronicity of ACL deficiency affects the tibiofemoral relationship in the sagittal plane, resulting in anterior translation of the tibia relative to the femur [16, 32]. Despite the extensive examination of anterior tibial translation (ATT) in the sagittal plane, there has been limited scrutiny of tibial rotation in the axial plane, while the tibia would theoretically also rotate in ACL‐deficient knees [3, 16, 19, 32].

The tibial tubercle‐trochlear groove (TT‐TG) distance, a well‐established parameter for evaluating patellar instability, reflects the amount of knee rotation [11, 35]. The TT‐TG distance was defined as the offset distance between the deepest point of the trochlear groove and the centre of the patellar tendon insertion on the tibial tuberosity assessed in the axial plane [17, 28]. Recent studies have identified the TT‐TG distance as a risk factor for both native ACL injury and graft failure following ACL reconstruction [14, 29]. However, to the best of our knowledge, no study has systematically investigated sequential tibiofemoral changes in the TT‐TG distance over time following ACL injury.

The objectives of this study were twofold: (1) to elucidate the changes in the tibiofemoral relationship over time following ACL injury, with a specific focus on the axial plane; and (2) to investigate the correlation between the tibiofemoral relationship and patient‐related outcome measures (PROMs). It was hypothesized that (1) the tibia would undergo anterior and internal translocation over time following an ACL injury, and (2) patients with significant translocation would experience worse PROMs.

## MATERIALS AND METHODS

This study was approved by the Institutional Review Board (approval number: 282‐215). Informed consent was obtained from all participants.

### Patients

**Figure 1:**
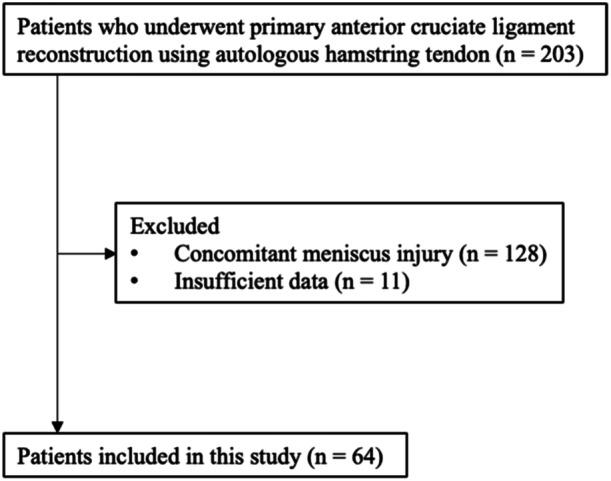
Flowchart of patient selection.

Between 2013 and 2022, 203 primary ACL reconstructions (ACLR) using autologous hamstring grafts were performed at our hospital. Patients who (1) underwent primary isolated ACLR, (2) undertook magnetic resonance imaging (MRI) within 1 year after trauma and (3) undertook computed tomography (CT) 1 week post‐operatively were included. We excluded patients with (1) meniscus injury, (2) unclear history of trauma, (3) multiple ligament injuries, (4) prior knee surgery and (5) insufficient data (Figure 1).

### Surgical technique and post‐operative rehabilitation

Anatomic triple‐bundle or double‐bundle ACLR was performed with autologous hamstring tendon grafts as described previously [30]. The meniscus was arthroscopically assessed during ACLR. The graft was fixed with Endobutton‐CL (Smith & Nephew Endoscopy) on the femur, whereas double‐spike plates (Meira Corp.) and screws were used on the tibial side, with an initial tension of 20 N at 15°–20° of knee flexion.

Post‐operatively, the knee was splint immobilized in a slightly flexed position. Range of motion exercises commenced 1 week post‐operatively. Partial weight bearing was permitted at 2 weeks, followed by full weight bearing at 3–4 weeks. Jogging was allowed at 3–4 months, and patients could return to sports activities between 6 and 8 months.

### Radiological evaluation

Pre‐operative MRI and post‐operative CT were performed on all patients. MRI images were obtained using a Canon Vantage 3.0‐Tesla and a quadrature detection coil (field of view, 160 mm; matrix, 368 × 224; slice thickness, 5.0 mm; interslice gap, 1 mm; receiver bandwidth, 224 kHz; repetition time, 500 ms; echo time, 10 ms). CT scans were obtained with the knee splint immobilized using a Canon Aquilion ONE (spiral scan, 0.5‐mm slice thickness).

**Figure 2:**
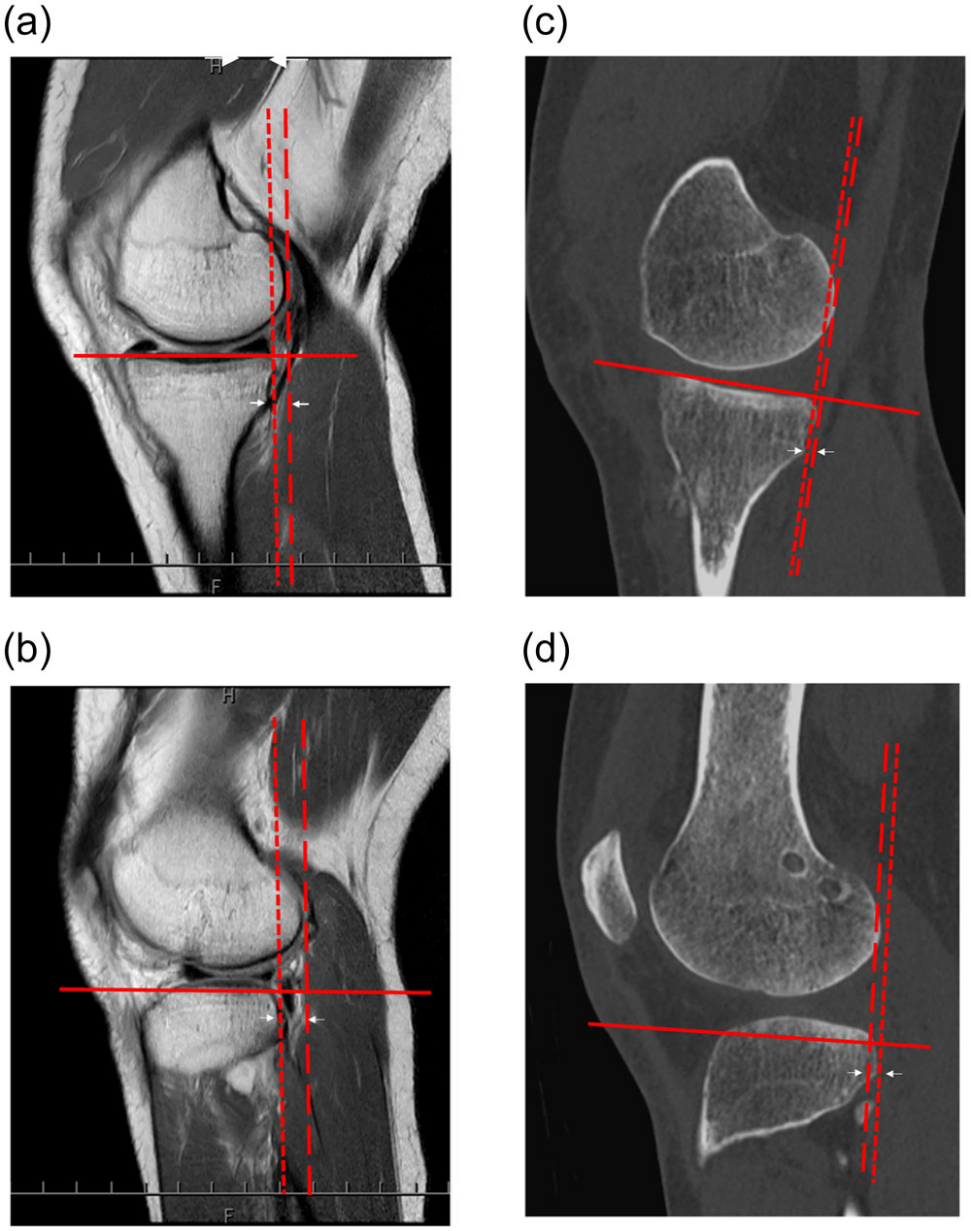
Measurement of the anterior tibial translation (ATT) on magnetic resonance imaging (MRI) and computed tomography (CT). The ATT is assessed using sagittal sequences. It is defined as the distance between the vertical line drawn to the posterior edge of the centre of each femoral condyle (dashed line) and the vertical line drawn to the posterior edges of each tibial plateau (dotted line). A positive value indicates the anterior translation of the tibia relative to the femur (arrow). (a) Measurement of medial ATT on MRI. (b) Measurement of lateral ATT on MRI. (c) Measurement of medial ATT on CT. (d) Measurement of lateral ATT on CT.

ATT was assessed using sagittal sequences (Figure 2). The ATT was defined as the distance between the vertical line drawn to the posterior edge of the femoral condyle and the vertical line drawn to the posterior edges of the tibial plateau [34]. A positive value indicated an anterior translation of the tibia relative to the femur.

**Figure 3:**
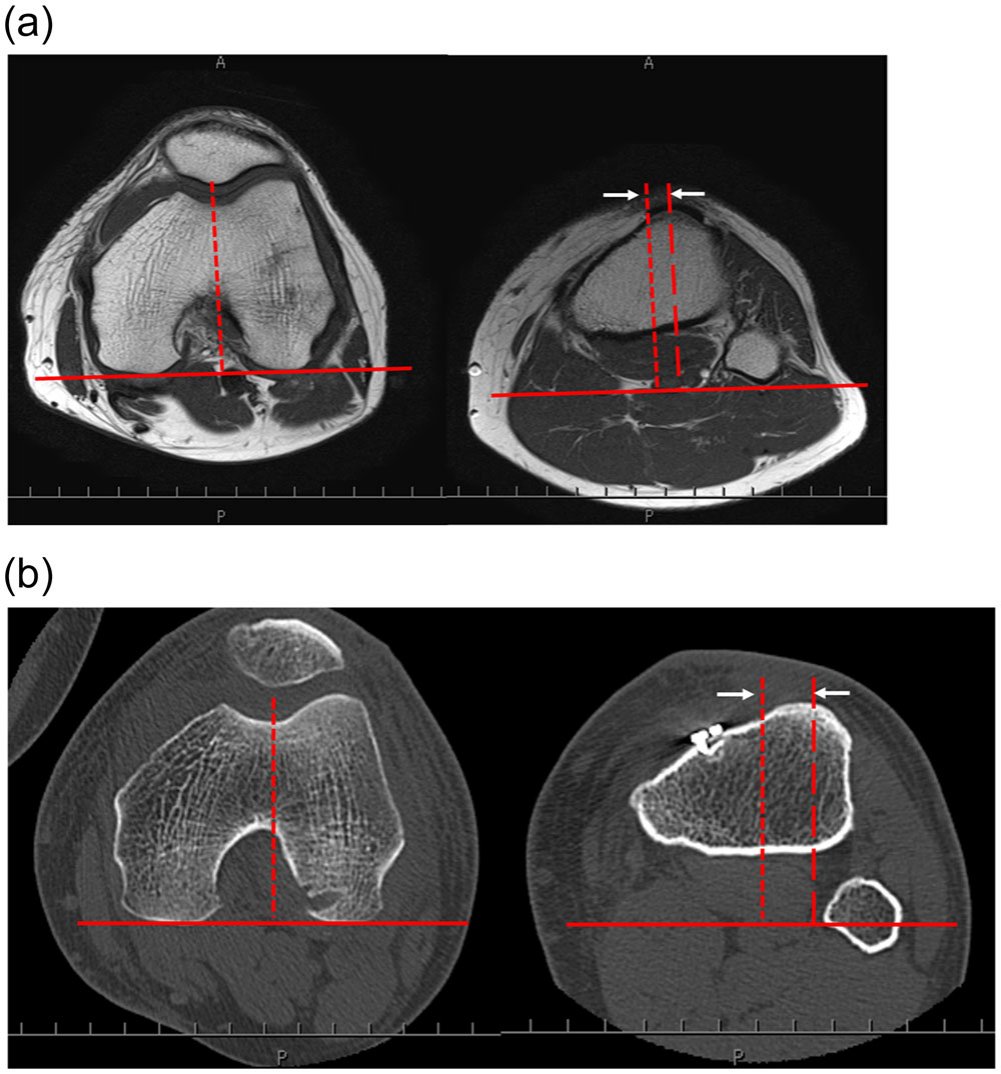
Measurement of tibial tubercle‐trochlear groove (TT‐TG) distance on magnetic resonance imaging (MRI) and computed tomography (CT). The TT‐TG distance is assessed using axial sequences. It is defined as the offset distance between the deepest point of the trochlear groove and the centre of the patellar tendon insertion on the tibial tuberosity. The most posterior point of the medial and lateral condyles of the femur is defined as the reference line (solid line). A line perpendicular to the reference line is drawn through the deepest bony point of the trochlear groove (dotted line). A second line perpendicular to the reference line is drawn through the most anterior point of the tibial tuberosity on the axial section, showing the most anterior point of the tuberosity (dashed line). The TT‐TG distance is defined as the distance between these two perpendicular lines (arrow). A positive value indicates external rotation of the tibia relative to the femur. (a) Measurement of TT‐TG distance on MRI. (b) Measurement of TT‐TG distance on CT.

TT‐TG distance, defined as the offset distance between the deepest point of the trochlear groove and the centre of the patellar tendon insertion on the tibial tuberosity, was assessed using axial sequences (Figure 3) [28]. The posterior point of the medial and lateral condyles of the femur was defined as the reference line. A line perpendicular to the reference line was drawn through the deepest bony point of the trochlear groove. A second line perpendicular to the reference line was drawn through the most anterior point of the tibial tuberosity on the axial section, showing the most anterior point of the tuberosity. The TT‐TG distance was defined as the distance between two perpendicular lines. A positive value indicated external rotation of the tibia relative to the femur.

Intraclass correlation coefficients (ICCs) were determined to assess the intra‐ and interobserver reproducibility. To assess intraobserver variations, all parameters were measured repeatedly by one observer 8 weeks later. To assess interobserver reliability, two independent blinded observers remeasured the subjects. The intra‐ and interobserver reliabilities for assessing radiological measurements showed substantial agreement, with ICC values >0.7.

The post‐operative medial ATT, lateral ATT and TT‐TG distances were established as baseline values for each patient. The difference between the pre‐operative and post‐operative values for each parameter was calculated: ΔMedial ATT, ΔLateral ATT and ΔTT‐TG distance. The correlation between each calculated value and the time elapsed since the ACL injury was assessed.

### Clinical evaluation

The clinical evaluation consisted of physical examination and PROMs. Knee joint laxity was assessed through the Lachman and pivot shift tests. The Lachman test was graded as negative, trace or positive. The pivot shift test was graded as negative, glide, clunk or gross, according to the International Knee Documentation Committee evaluation form [12]. Anterior laxity was quantified using a KT‐1000 arthrometer (MED Metrics). PROMs were assessed using the Knee Injury and Osteoarthritis Outcome Score (KOOS) [26]. The correlation between each calculated value and the KOOS subscales was assessed.

### Statistical analysis

Statistical analyses were performed using EZR (version 1.61; Saitama Medical Center, Jichi Medical University), a graphical interface for R (version 4.2.2.; R Foundation for Statistical Computing) [13].

Correlations were examined using Spearman's rank correlation test. Continuous variables were analysed using a paired *t* test. Statistical significance was defined as *p* values <0.05.

A priori power analysis was performed to determine an appropriate sample size using G*Power version 3.1.0 (Heinrich Heine University, Duesseldorf, Germany). A sample size of 42 patients would provide a statistical power of 0.95 at a significance level (alpha) of 0.05, with an effect size of 0.50.

## RESULTS

**Figure 4:**
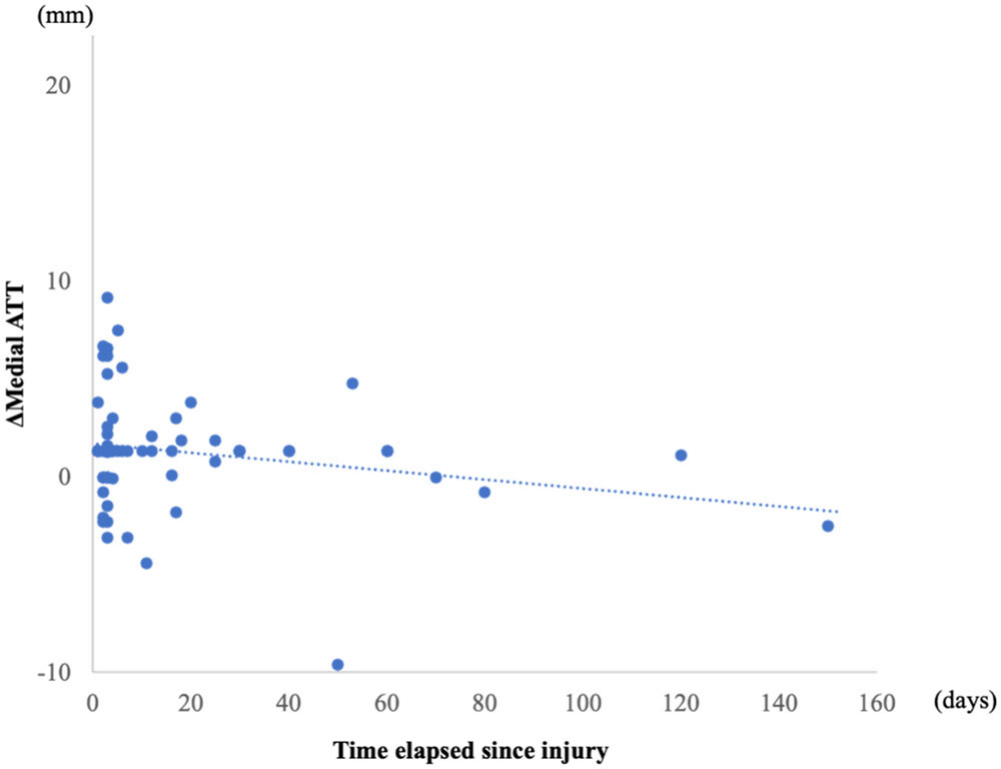
Correlation between the change in medial anterior tibial translation (ΔMedial ATT) and time elapsed since injury (*r* = −0.04, *p* = 0.78).

**Figure 5:**
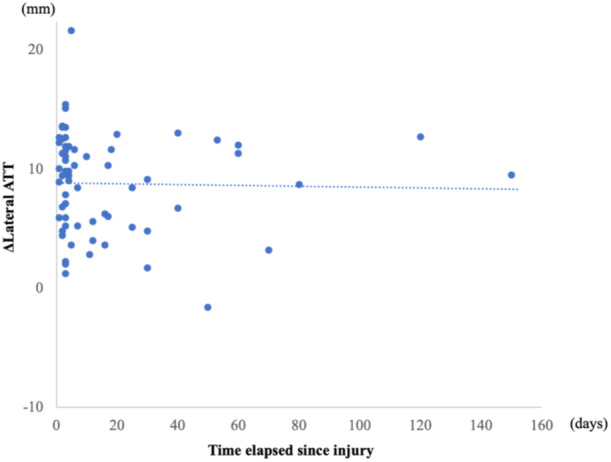
Correlation between the change in lateral anterior tibial translation (Δ Lateral ATT) and time elapsed since injury (*r* = −0.15, *p* = 0.25).

**Figure 6:**
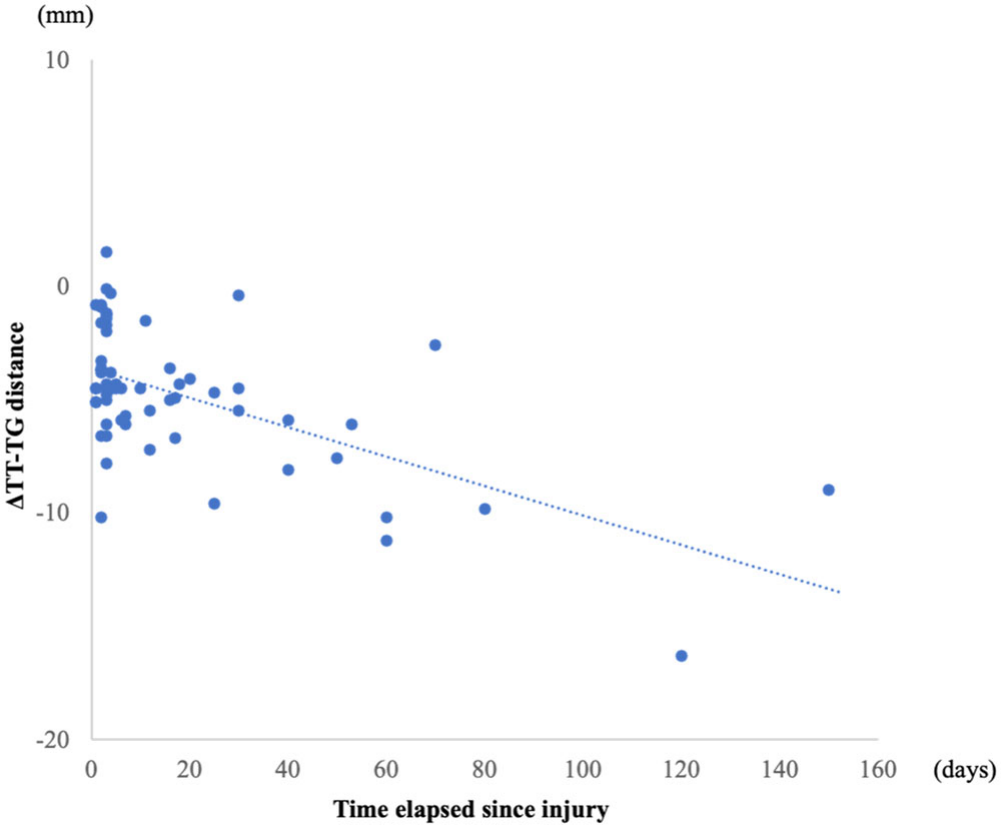
Correlation between the change in the tibial tubercle‐trochlear groove (TT‐TG) distance (ΔTT‐TG distance) and time elapsed since injury (*r* = −0.44, *p* < 0.01).

Sixty‐four patients were enroled in this study; their demographic characteristics are shown in Table 1. The medial ATT, lateral ATT and TT‐TG distances were significantly different pre‐operatively compared to post‐operative values (*p* < 0.01 for all), with the tibia translated anteriorly and rotated internally relative to the femur (Table 2). The ΔMedial ATT and ΔLateral ATT were 1.6 and 8.8 mm, respectively. No correlation was found between ΔMedial ATT and the time elapsed since the injury, nor between ΔLateral ATT and the time elapsed since the injury (Figures 4 and 5). The ΔTT‐TG distance was −4.8 mm. A negative correlation was observed between the ΔTT‐TG distance and the time elapsed since the injury (*r* = −0.44, *p* < 0.01) (Figure 6). Table 3 provides a summary of post‐operative physical examinations. All KOOS subscale scores improved significantly post‐operatively (Table 4). Neither the ΔMedial ATT, ΔLateral ATT, nor ΔTT‐TG distance correlated with the pre‐operative or post‐operative KOOS subscale scores.

**Table 1:** Patient characteristics.

Age, years	26.7 ± 14.0
Sex, *n*, male/female	35/29
BMI, kg/m^2^	22.5 ± 3.8
Time elapsed from injury to MRI, days	17.7 ± 28.3
Follow‐up period, months	27.6 ± 18.0

*Note*: All data expressed as mean ± standard deviation

Abbreviations: BMI, body mass index; MRI, magnetic resonance imaging.

**Table 2:** Imaging measurements.

	Pre‐operative	Post‐operative	*Δ*	*p* Value
Medial ATT, mm	0.4 ± 2.8	−1.2 ± 2.4	1.6 ± 2.6	<0.01
Lateral ATT, mm	5.3 ± 4.3	−3.5 ± 3.6	8.8 ± 4.2	<0.01
TT‐TG distance, mm	9.7 ± 3.2	14.4 ± 2.8	−4.8 ± 3.1	<0.01

*Note*: All data expressed as mean ± standard deviation.

Abbreviations: ATT, Anterior tibial translation; TT‐TG, tibial tuberosity‐trochlea groove.

**Table 3:** Post‐operative physical examinations.

Lachman test, negative/trace/positive	62/2/0
Pivot shift test, negative/glide/clunk/gross	58/6/0/0
KT‐1000, mm	0.9 ± 0.7

**Table 4:** Pre‐operative and post‐operative KOOS.

	Pre‐operative	Post‐operative	*p* Value
Symptom	73.4 ± 21.9	87.7 ± 12.1	<0.01
Pain	77.5 ± 21.9	94.1 ± 8.0	<0.01
ADL	79.1 ± 25.9	97.7 ± 3.4	<0.01
Sports/recreation	49.2 ± 27.9	89.9 ± 14.1	<0.01
QOL	51.1 ± 26.1	85.2 ± 17.3	<0.01

*Note*: All data expressed as mean ± standard deviation.

Abbreviations: ADLs, activities of daily life; KOOS, Knee Injury and Osteoarthritis Outcome Score; QOL, quality of life.

## DISCUSSION

The principal finding of this study is that the tibia underwent internal rotation relative to the femur over time following an ACL injury, despite the absence of a correlation in anterior tibial translation. Nevertheless, pre‐operative medial ATT, lateral ATT and TT‐TG distances were significantly different compared to post‐operative values, with the tibia translating anteriorly and rotating internally relative to the femur after ACL injury.

The ACL, originating from the anteromedial aspect of the tibial plateau to the medial wall of the lateral femoral condyle, plays a crucial role in maintaining a normal tibiofemoral relationship. When the ACL ruptures, it is estimated that the tibia translates anteriorly and rotates internally relative to the femur, although most previous studies have primarily focused on the ATT in the sagittal plane [3, 16]. In studies investigating ATT following ACL injury, the extent of ATT has been shown to be influenced by the chronicity of ACL deficiency, resulting in anterior translation of the tibia relative to the femur [15, 16, 32]. However, no significant correlation was observed between the ATT and the time elapsed since the ACL injury in this study. This may be explained by the fact that this study included patients who underwent MRI within 1 year of ACL injury, whereas ATT has been reported to increase more than 2 years after the ACL injury [18, 32]. To prevent statistical bias towards a specific value, patients with more than 1 year elapsed from injury to undergoing MRI were excluded, as only a few patients visited our hospital after primary ACL injury. Additionally, the exclusion of patients with meniscus injuries, which can influence knee joint laxity, may have impacted our results [6, 21].

Many studies have evaluated the tibiofemoral relationship after ACL injury [4, 8, 15, 32]. The lateral monopodal weight‐bearing radiograph is an easy and reproducible method to measure ATT under loading conditions [4]. Recently, measuring the side‐to‐side difference in ATT under loading conditions in both healthy and ACL‐deficient knees has been implemented as a part of the follow‐up of ACL‐injured patients [15]. Monopodal weight‐bearing radiography offers an easy and objective assessment method. However, several patients could not assume a monomodal position owing to pain and knee instability immediately after ACL injury. Additionally, patients were typically allowed full weight‐bearing 3–4 weeks after ACL reconstruction. Therefore, in our study, MRI and CT were used to evaluate ATT.

Notably, our results represent the first published report identifying an association between the chronicity of ACL deficiency and tibial rotation, as represented by the TT‐TG distance. Several studies have suggested that an increased TT‐TG distance, indicative of external rotation of the tibia, is associated with ACL injury or graft failure after ACL reconstruction [2, 27, 29]. It is conceivable that a larger TT‐TG distance increases the anterior shear force on the tibia, consequently imposing more stress on the ACL [29]. However, in an ACL‐deficient knee, the tibia theoretically experiences internal rotation relative to the femur, resulting in a decrease in the TT‐TG distance [1]. In this study, with respect to the association between the chronicity of ACL deficiency and the TT‐TG distance, the amount of tibial internal rotation was larger in patients with a longer time elapsed from the injury to undergoing MRI, which was theoretically valid.

Regarding clinical outcomes, no significant correlation was found between pre‐operative and post‐operative PROMs and the extent of tibial translation or rotation subsequent to ACL injury. One potential explanation for this lack of correlation is the intentional exclusion of patients with meniscus injuries in our study, as concurrent meniscus injuries might influence clinical outcomes after ACL injury. Furthermore, a previous study highlighted the association between an abnormal pre‐operative tibiofemoral relationship and early graft failure, focusing on the ATT in the sagittal plane [33]. Nevertheless, the rotational aspect of tibial translocation after an ACL injury may also affect early graft failure. Consequently, considering the potential influence of abnormal tibiofemoral relationships caused by meniscus injury and soft tissue decompensation, early ACLR should be considered as a preventive measure.

The strength of this study lies in the exclusion of patients with concomitant meniscus injuries to eliminate the influence of the meniscus on knee joint laxity. This approach sets our study apart from most previous studies, which included patients with concomitant meniscus injuries [6, 21, 24, 32]. It remains uncertain whether an abnormal tibiofemoral relationship resulting from an ACL injury precedes a meniscus injury, or if a concomitant meniscus injury with an ACL injury induces an abnormal tibiofemoral relationship [5, 7, 25]. However, it is widely acknowledged that the meniscus plays a crucial role in the load‐bearing distribution and contributes to joint stabilization [6, 7, 9]. Consequently, patients with meniscus injuries were deliberately excluded from this study to assess the correlation between the time elapsed since the ACL injury and the tibiofemoral relationship.

This study had several limitations. First, MRI and CT were performed only on the injured side. Considering individual differences in anatomy, with variations in the ATT and TT‐TG distance among individuals, a side‐to‐side comparison might have been more informative. However, ethical, social and economic constraints prevented us from conducting MRI and CT of both knees. On the other hand, anatomical ACLR has been proven to restore normal kinematics of the knee joint [16, 22, 23, 31, 32]. Similarly, our post‐operative physical examination and clinical scores showed satisfactory outcomes. In addition, all patients underwent ACLR in the same manner in a single centre. Therefore, the post‐operative measurement values were defined as a baseline for each patient, and the difference between pre‐operative and post‐operative values was used to assess the correlations. Second, CT was taken pre‐operatively, whereas MRI was taken post‐operatively. Lower TT‐TG distances on MRI compared with CT were reported, which depended on the imaging protocol technique within each centre. Specifically, it was also assumed that patient positioning, rather than factors intrinsic to CT or MRI modality, was likely responsible for measurement discrepancies [10]. In this study, CT and MRI were taken with the same protocol in a single centre. Additionally, post‐operative CT scans were obtained with the knee splint immobilized, whereby the knee position was similar to that of a fixed knee coil for MRI; we believe that this lessens the variability depending on patient positioning.

## CONCLUSION

The principal finding of this study is that the tibia undergoes internal rotation relative to the femur over time following an ACL injury, despite the absence of a correlation in anterior tibial translation. While the pre‐operative and post‐operative PROMs did not correlate with the extent of anterior tibial translation or internal rotation subsequent to ACL injury, the chronicity of ACL deficiency might lead to a meniscus injury, resulting in worse clinical outcomes. These findings emphasize the importance of early consideration of ACLR to prevent abnormal tibiofemoral relationships, providing valuable insights for clinicians managing patients with ACL injuries.

## AUTHOR CONTRIBUTIONS

All authors (1) made substantial contributions to the study concept, data analysis and interpretation; (2) drafted the manuscript or revised it critically for important intellectual content; (3) approved the final version of the manuscript to be published and (4) agreed to be accountable for all aspects of the work.

## FUNDING INFORMATION

The authors did not receive support from any organization for the submitted work.

## CONFLICT OF INTEREST STATEMENT

The authors declare no conflict of interest.

## ETHICS STATEMENT

This study was approved by the Institutional Review Board (approval number: 282‐215). This retrospective chart review involving human participants was conducted in accordance with the ethical standards of the Institutional Committee and the 1964 Declaration of Helsinki and its later amendments or comparable ethical standards. All study participants provided informed consent, and the study design was approved by the appropriate ethics review board.

## Data Availability

Due to the nature of this research, participants of this study did not agree for their data to be shared publicly, so supporting data are not available.
